# The development of *L*. *major*, *L*. *donovani* and *L*. *martiniquensis*, *Leishmania* currently emerging in Europe, in the sand fly species *Phlebotomus perniciosus* and *P*. *tobbi*

**DOI:** 10.1371/journal.pntd.0012597

**Published:** 2024-10-15

**Authors:** Jovana Sadlova, Anna Hoskova, Barbora Vojtkova, Tomas Becvar, Petr Volf

**Affiliations:** Department of Parasitology, Faculty of Science, Charles University, Prague, Czechia; Aix-Marseille Universite, FRANCE

## Abstract

**Background:**

Several new species of *Leishmania* have recently emerged in Europe, probably as the result of global changes and increased human migration from endemic areas. In this study, we tested whether two sand fly species, the Western Mediterranean *Phlebotomus perniciosus* and the Eastern Mediterranean *P*. *tobbi*, are competent vectors of *L*. *donovani*, *L*. *major* and *L*. *martiniquensis*.

**Methodology/principal findings:**

Sand flies were infected through the chick skin membrane using *Leishmania* species and strains of various geographical origins. *Leishmania* infections were evaluated by light microscopy and qPCR, and the representation of morphological forms was assessed from Giemsa-stained gut smears. Neither *P*. *perniciosus* nor *P*. *tobbi* supported the development of *L*. *martiniquensis*, but *L*. *major* and *L*. *donovani* in both species survived defecation of blood meal remnants, colonized the stomodeal valve and produced metacyclic stages. The results with *L donovani* have shown that infection rates in sand flies can be strain-specific; therefore, to determine vector competence or refractoriness, it is optimal to test at least two strains of *Leishmania*.

**Conclusions, significance:**

Both sand fly species tested are potential vectors of *L*. *donovani* and *L*. *major* in Mediterranean area. However, further studies will be needed to identify European vectors of *L*. *martiniquensis* and to test the ability of other European sand fly species to transmit *L*. *major*, *L*. *donovani*, *L*. *tropica* and *L*. *infantum*.

## Author summary

Leishmaniases are serious human and animal diseases caused by protozoa of the genus *Leishmania* (Kinetoplastida: Trypanosomatidae), transmitted by blood-feeding insects – phlebotomine sand flies (Diptera: Psychodidae). They are mainly distributed in tropical and subtropical regions, but recently the ranges of the vectors have spread more into temperate regions. In Europe, species of *Leishmania* that were previously absent have emerged, mainly due to the mass migration of the refugees from endemic areas. It is therefore important to know whether local sand flies can transmit these “new” *Leishmania* species. In our study, we tested two widely distributed sand fly species, *P*. *perniciosus* and *P*. *tobbi*. In both of them, *L*. *major* and *L*. *donovani* completed their development, thus *P*. *perniciosus* and *P*. *tobbi* are potential vectors of these parasites. The third *Leishmania* species tested, *L*. *martiniquensis*, which has been repeatedly recorded in Central Europe, did not survive in *P*. *perniciosus* and *P*. *tobbi*; its vectors have yet to be confirmed.

## 1. Introduction

Leishmaniases are human and animal vector-borne diseases caused by parasitic protozoans of the genus *Leishmania* (Kinetoplastida: Trypanosomatidae) endemic in 99 countries on five continents, mainly in tropical and subtropical countries, with an estimated 700 000 to 1 million new cases occurring annually [1]. The increasing trend in the number of new cases has been observed in the last decade, mainly due to the outbreak in the Eastern Mediterranean region [2]. Political and social instability in this region and adjacent areas led to thousands of refugees migrating to Europe every year. Indeed, leishmaniasis is the most frequently reported infectious disease among migrants from Syria [3]. Thus, this trend, together with ongoing climate change, pose potential risk of introduction of new leishmania species and the need for a close monitoring of the incidence of leishmaniases in European countries [4,5].

In southern Europe, *L*. *infantum* has been endemic since ancient times and is currently found in all Mediterranean and Balkan countries [6]. Among other *Leishmania* species, only local transmission of *L*. *tropica* has been sporadically reported from Greece [7]. However, the situation changes and two other *Leishmania* species are now emerging in Europe, *L*. *donovani* causing both visceral (VL) and cutaneous leishmaniasis (CL) and *L*. *major* causing CL. In Cyprus, human cases of *L*. *donovani sensu stricto* were first detected in 2006 [8] and a local transmission cycle was confirmed a decade later [9]. In southern Turkey, *L*. *major* and *L*. *donovani* were first reported as imported cases from Syria and Iraq in 2014 [10], and then autochthonous cases also appeared here several years later [11,12]. In Portugal, *L*. *major* has been detected in naturally infected sand flies of the species *Sergentomyia minuta* [13], in stray cats [14] and as a hybrid with *L*. *infantum* in HIV-positive patients [15]. Moreover, the epidemiological situation of European leishmaniases has recently become even more complicated with the discovery of *Leishmania* (*Mundinia*) species, *L*. *martiniquensis*, in horses and cattle in Central Europe [16,17].

The predominant vectors of *Leishmania* are phlebotomine sand flies (Diptera: Psychodidae) [18]. Recently, their geographical range has expanded in Europe to more northern latitudes due to ongoing climatic and environmental changes-*P*. *mascittii* and *P*. *perniciosus* were first detected in Germany in 1999 and 2001, respectively [19,20], *P*. *mascitti* was later recorded also in Austria [21] and Slovakia [22] and a few years ago, *P*. *simici* was found in Austria [23]. Consequently, autochthonous cases of *L*. *infantum* infections have been reported since 2001 in children and horses in southern Germany and Austria [24,25].

Several representatives of the subgenus *Larroussius* are proven vectors of *L*. *infantum* in Europe: *Phlebotomus perniciosus*, *P*. *ariasi*, *P*. *perfiliewi*, *P*. *neglectus* and *P*. *tobbi* [26]. All *Larroussius* species tested so far have proven to be permissive vectors [27], thus supporting the development of a wider range of *Leishmania* species [28–30]. Nevertheless, susceptibility of these European sand flies to the *Leishmania* species currently spreading to Europe (*L*. *donovani*, *L*. *major*, *L*. *martiniquensis*) is understudied. Therefore, here we tested the vector competence of *P*. *perniciosus* (widespread in the Western Mediterranean) and *P*. *tobbi* (widespread in the Eastern Mediterranean), to complete fragmentary data on this topic using *Leishmania* species and strains of various geographical origins.

## 2. Materials and methods

### 2.1. Sand flies and *Leishmania*

Laboratory colonies of *P*. *perniciosus* (originally from Spain) and *P*. *tobbi* (originally from Turkey) were maintained in the insectary of the Charles University in Prague under standard conditions (26°C, fed on 50% sucrose and photoperiod 14 h light/ 10 h dark) as described previously [31].

*Leishmania donovani* MHOM/NP/03/BPK282, *L*. *infantum* MHOM/TR/2000/OG-VL, *L*. *infantum/L*. *donovani hybrid* ITOB/TR/2005/CUK3, *L*. *major* MHOM/LY/87/CS3 and *L*. *martiniquensis* MHOM/TH/2019/Cu2 and MEQU/CZ/2019/Aig1 were cultured in M199 medium (Sigma) containing 10% heat-inactivated fetal calf serum (Gibco) supplemented with 2% sterile urine, 1% BME vitamins (Basal Medium Eagle, Sigma) and 250 μg/ml amikacin (Medochemie LTD).

### 2.2. Experimental infections of sand flies

*Leishmania* promastigotes from log-phase cultures were resuspended in heat-inactivated defibrinated ram blood at a concentration of 1 × 10^6^ promastigotes/ml. Female sand flies were allowed to feed through a chick-skin membrane and engorged individuals were maintained in the same conditions as the colony for subsequent dissections at day 2, 4 and 10 post bloodmeal (PBM). The intensity and localization of infections were evaluated under the light microscope; the infections were scored as light (<100 parasites per gut), moderate (100–1000 parasites per gut) and heavy (>1000 parasites per gut) according to [32]. Estimating the number of *L*. *martiniquensis* was difficult in engorged females on day 2 PBM because the small promastigotes with a rounded body and very short flagella were barely distinguishable from red blood cells. Therefore, the infection rate at this time point was estimated from Giemsa-stained gut smears.

### 2.3. Morphological analysis of parasites

Morphology of parasites in mature infections was evaluated from methanol-fixed and Giemsa-stained gut smears obtained on day 10 PBM. Promastigotes were examined by light micro- scopy with an oil immersion objective and photographed using the Olympus DP70 camera. The body length, body width and flagellum length of the parasites were measured using Image J software. Promastigotes were scored as metacyclic forms when flagellum length ≥ 2 times body length, leptomonad forms when flagellum length < 2 times body length and body length was < 14 μm, and elongated nectomonads when flagellum length < 2 times body length and body length ≥ 14 according to [33]. Haptomonads characterized by a disc-shaped extension of the flagellar tip were recorded but not quantified because these forms, attached to the sand fly gut, are underestimated on gut smears.

### 2.4. PCR quantification of parasites

DNA extraction from insect tissues was performed using the High Pure PCR Template Preparation Kit (Roche Diagnostics, Indianapolis, IN) according to the manufacturer’s instructions. The qPCR for detection and quantification of *Leishmania* was performed in Bio-Rad iCycler & iQ Real-Time PCR Systems using the SYBR Green detection method (iQ SYBR Green Supermix, Bio-Rad, Hercules, CA) as described previously [34] using the kinetoplast minicircle primers (forward primer 5′-CTTTTCTGGTCCTCCGGGTAGG-3′ and reverse primer 5′-CCACCCGGCCCTATTTTA CACCAA-3′) [35].

### 2.5. Statistical analysis

Differences in infection rate and the proportion of morphological forms were analysed by Pearson’s Chi-square test (multi-field tables) or Fisher’s test (four-field tables). If N<30 or if >20% of cells had expected count less than 5, exact variants of the tests were used and if zeros were present, Monte Carlo simulation was used; differences in the representation of each category between species were tested by z-test. Nonparametric Median test and Kruskal-Wallis test were used to analyze the number of promastigotes detected by qPCR. All statistical analyses were performed using SPSS version 27.

## 3. Results

### 3.1 Development of *L*. *donovani*, *L*. *major* and *L*. *martiniquensis* in *P*. *perniciosus*

**Fig 1:**
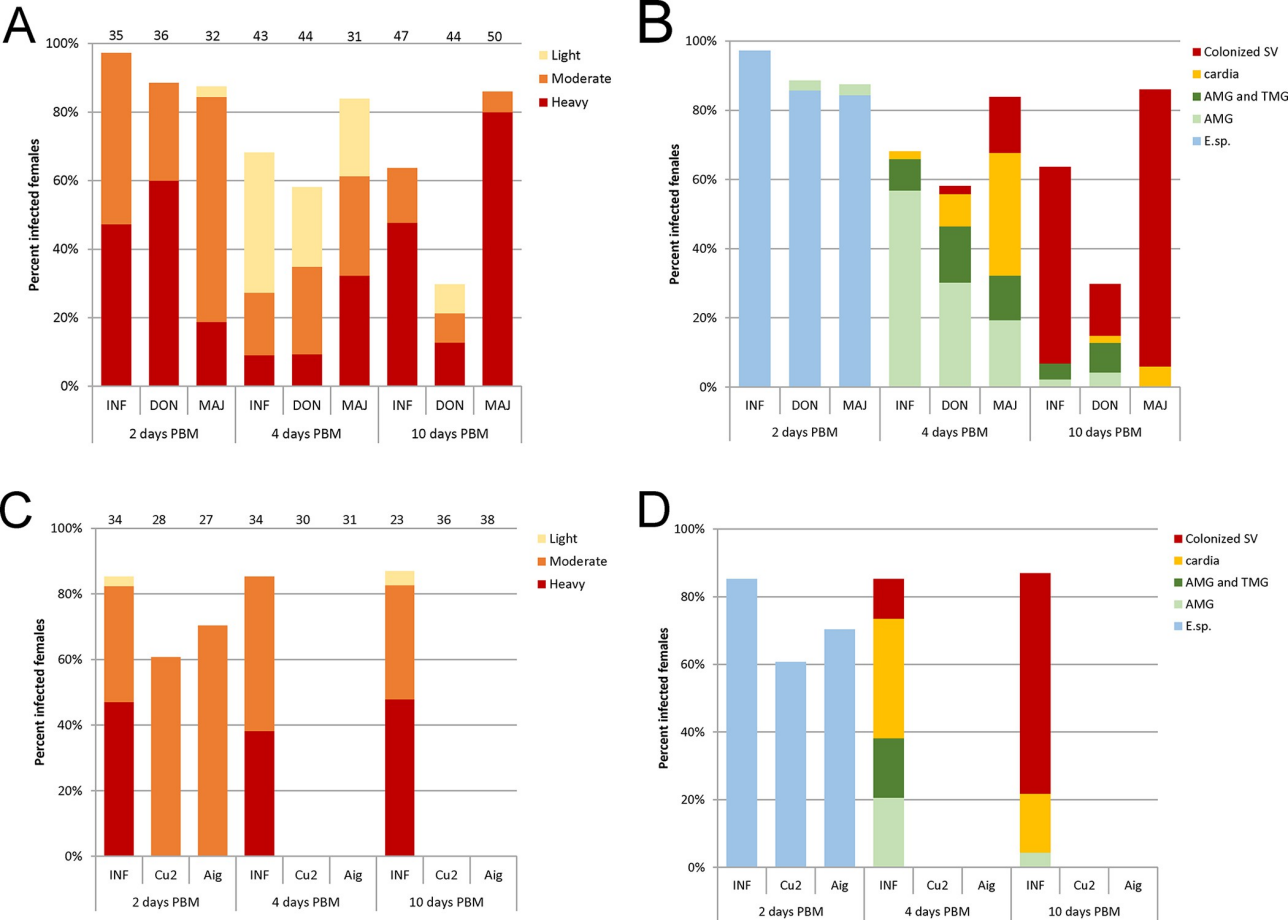
*Leishmania* infections in *P*. *perniciosus* females. A, C, intensities of infections; B, D, localisation of infections. A, B, summary of 3 experiments with *Leishmania donovani* (DON) and *L*. *major* (MAJ); C, D, summary of 2 experiments with *L*. *martiniquensis* strain Cu2 (Thailand) and strain Aig (Czechia). *Leishmania infantum* (INF) was used as a positive control in all experiments. The numbers of females examined are shown above the columns of infection intensities. SV, stomodeal valve; AMG, abdominal midgut; TMG, thoracic midgut; E. Sp., endoperitrophic space.

In the first series of experiments, *P*. *perniciosus* females were infected with the Nepalese isolate of *L*. *donovani* BPK282 and the Libyan isolate of *L*. *major* CS3; *L*. *infantum* isolated from a patient in Turkey (OG-VL) served as a positive control. On the second day PBM, prior to defecation, the infection rate was higher than 87% with no significant differences between groups (Fig 1A; P = 0.258, Χ^2^ = 2.635). *Leishmania* were present in the endoperitrophic space of most dissected females, only rarely were observed outside the peritrophic matrix in the abdominal midgut (Fig 1B).

On the fourth day PBM, the highest infection rate was observed in females infected with *L*. *major*, but differences to the other groups were not significant (Fig 1A; P = 0,065, d.f. = 2, Χ^2^ = 5,548). In females infected with *L*. *infantum* and *L*. *donovani*, abdominal midgut infections predominated, while promastigotes of *L*. *major* were largely found in the foregut and had already colonized the stomodeal valve in 16% of females (Fig 1B).

**Fig 2:**
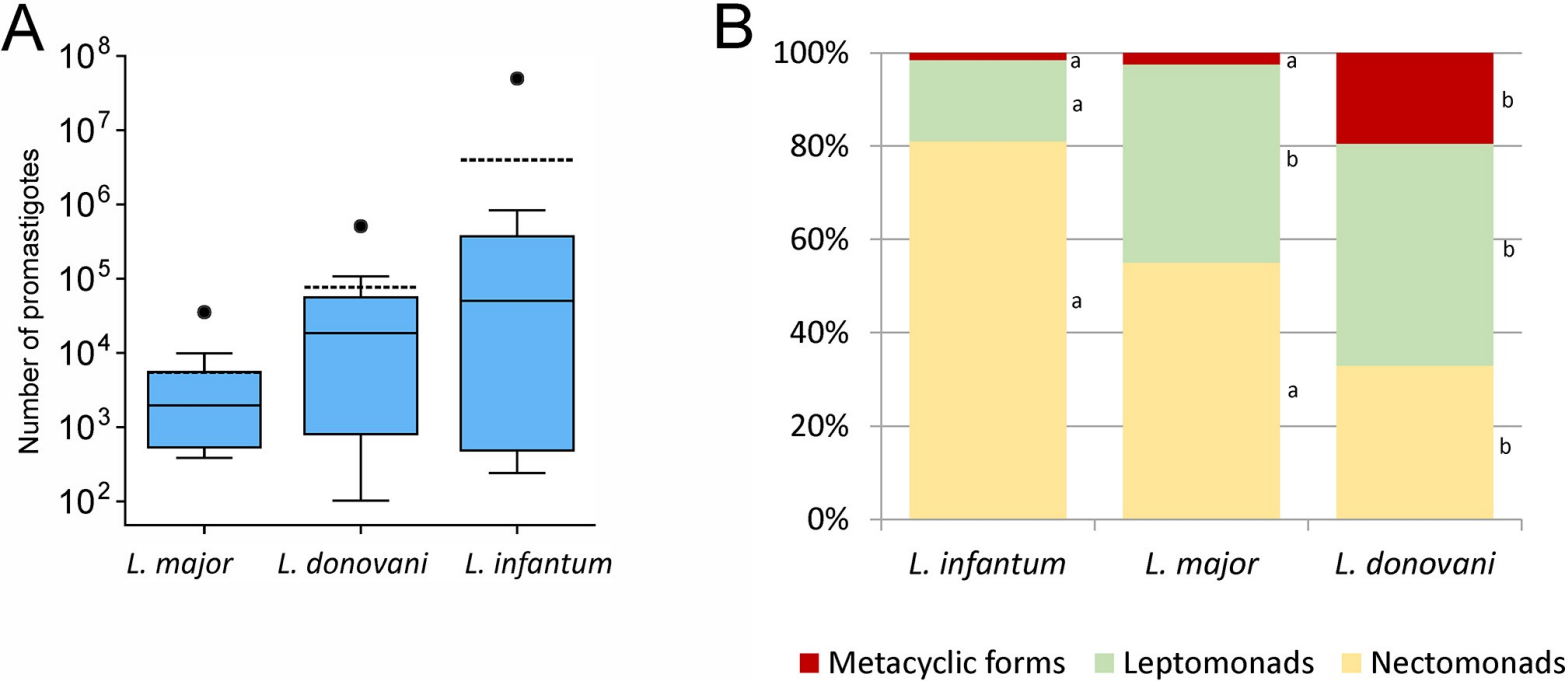
Numbers and morphology of *Leishmania* promastigotes in midguts of *P*. *perniciosus* on day 10 PBM. A, Parasite load in sand flies infected with *L*. *major* (N = 12), *L*. *donovani* (N = 10) and *L*. *infantum* (N = 13); the original data available in S1 Dataset. In the boxplots, the box is bordered by upper and lower quartile (IQR, interquartile range), the horizontal full line denotes the median value, the horizontal dashed line shows the mean, whiskers denote 1.5 times IQR and circles denote outliers. B, Representation of morphological forms in 200 promastigotes of each species. Small letters to the right of the columns indicate the significance of differences in the representation of morphological forms: if the letters are the same, the representation of forms does not differ for these *Leishmania* species; the original data available in S2 Dataset.

On day 10 PBM, all *Leishmania* species formed mostly heavy mature infections and colonized the stomodeal valve (Fig 1B). At this time, the infection rates varied considerably between groups, being significantly lower in sand flies infected with *L*. *donovani* and higher in those infected with *L*. *major* compared to the control group infected with *L*. *infantum* (Fig 1A; P < 0.001, d.f. = 2, Χ^2^ = 32.274). Parasite numbers in mature infections (day 10 PBM) were compared also using qPCR (Fig 2A). The highest numbers of parasites were detected in the control *L*. *infantum*, but the differences in median values and their distribution were not significant (Nonparametric Median test P = 0.098; Kruskal-Wallis test P = 0.243). Morphological analysis of promastigotes from gut smears showed that all the species form metacyclic stages in mature infections (Fig 2B). The proportion of metacyclic stages was significantly higher in *L*. *donovani* than in both *L*. *major* and control *L*. *infantum* (P < 0.001, d.f. = 4, Χ^2^ = 122.087).

To test the development of *L*. *martiniquensis* in *P*. *perniciosus*, two strains were used; one isolated from a human in Thailand (Cu2) and the other from a horse in the Czechia (Aig). The same strain of *L*. *infantum* served as a positive control as in the previous experiments. On the second day PBM, infections were observed in 60.7% of females infected with the Thai strain and 70.4% of females infected with the Czech strain (Fig 1C) with no statistically significant difference against the *L*. *infantum* control group (P = 0.097, d.f. = 2, Χ^2^ = 4.85). *Leishmania* occurred in the endoperitrophic space in all three groups (Fig 1D). However, at later time intervals, no infected females were found with either strain of *L*. *martiniquensis*, while the control *L*. *infantum* developed normally, promastigotes formed heavy infections and colonized the stomodeal valve (Fig 1C and 1D). This indicates that *L*. *martiniquensis* did not survive defecation of *P perniciosus* females and failed to develop mature infections; thus *P*. *perniciosus* is not a competent vector of this *Mundinia* species.

### 3.2. Development of *L*. *donovani*, *L*. *major* and *L*. *martiniquensis* in *P*. *tobbi*

For *P*. *tobbi* infections, the hybrid *L*. *infantum*/*L*. *donovani* strain CUK3 was used as a positive control as it was isolated from this sand fly species in Turkey [36]. Strains of *L*. *donovani*, *L*. *infantum* and *L*. *martiniquensis* were the same as in the experiments with *P*. *perniciosus*.

**Fig 3:**
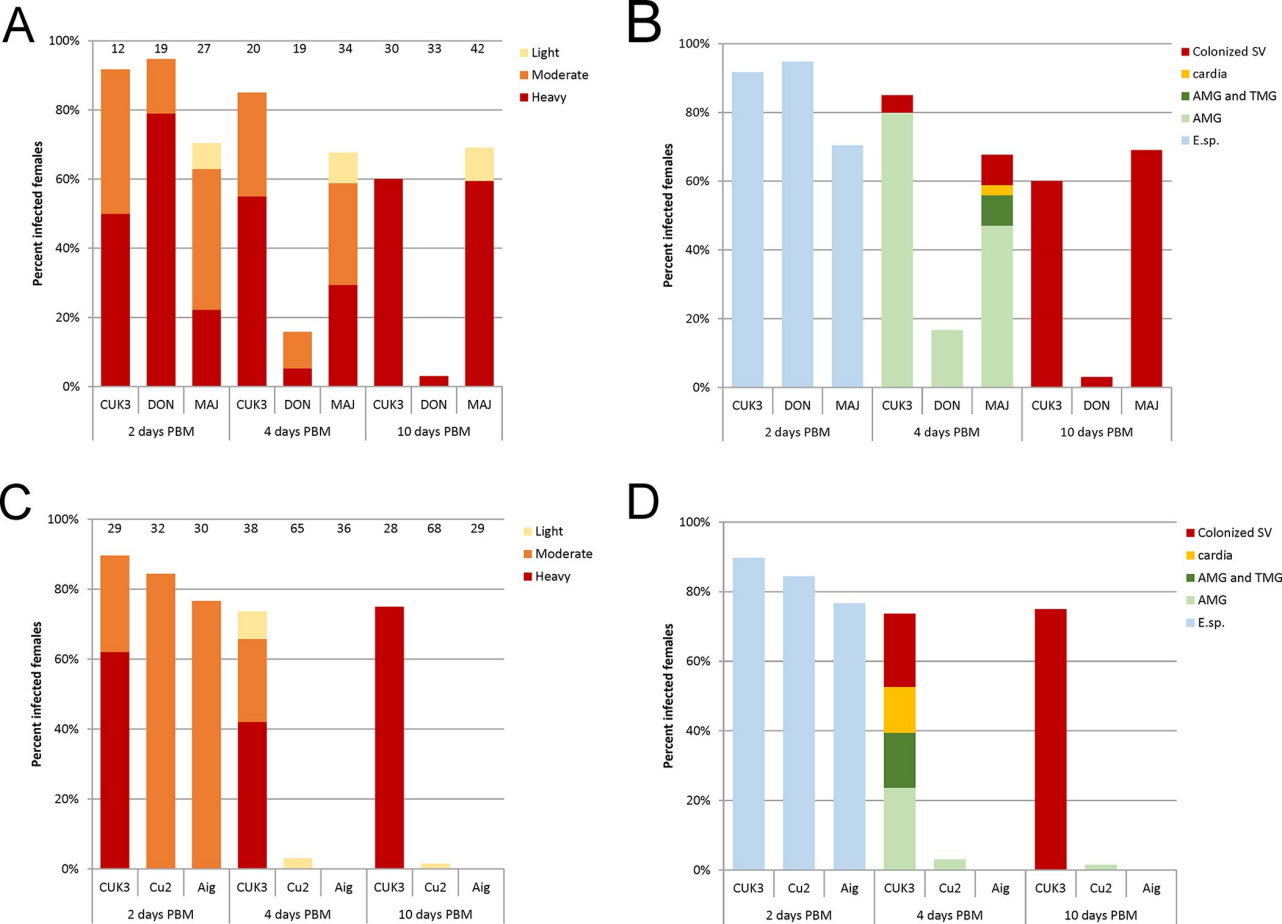
*Leishmania* infections in *P*. *tobbi* females. A, C, intensities of infections; B, D, localisation of infections. A, B, summary of 5 experiments with *Leishmania donovani* (DON) and *L*. *major* (MAJ); C, D, summary of 4 experiments with *L*. *martiniquensis* strain Cu2 (Thailand) and strain Aig (Czechia). *Leishmania infantum/L*. *donovani* hybrid (CUK3) was used as a positive control in all experiments. The numbers of females examined are shown above the columns of infection intensities. SV, stomodeal valve; AMG, abdominal midgut; TMG, thoracic midgut; E. Sp., endoperitrophic space.

Two days PBM, *L*. *donovani* and *L*. *major* developed similar infection rates in *P*. *tobbi* females as the control *L*. *infantum* (Fig 3A; P = 0,079, Χ^2^ = 4,825). *Leishmania* occurred in the endoperitrophic space in females of all groups (Fig 3B). On day 4 PBM, the proportion of infected females in the *L*. *donovani*-infected group was significantly lower compared to the other groups (Fig 3A; P < 0.001, d.f. = 2, Χ^2^ = 21.29). They occurred only in the abdominal midgut, while *L*. *major* was already present in the thoracic midgut and cardia and in 3 females it even colonized the stomodeal valve, as in the control *L*. *infantum* (Fig 3B). On day 10 PBM, heavy infections occurred and *Leishmania* colonized the stomodeal valve in all three groups but the infection rate of *L*. *donovani* was significantly lower compared to the other groups (Fig 3A and 3B; P < 0.001, d.f. = 2, Χ^2^ = 35.909).

**Fig 4:**
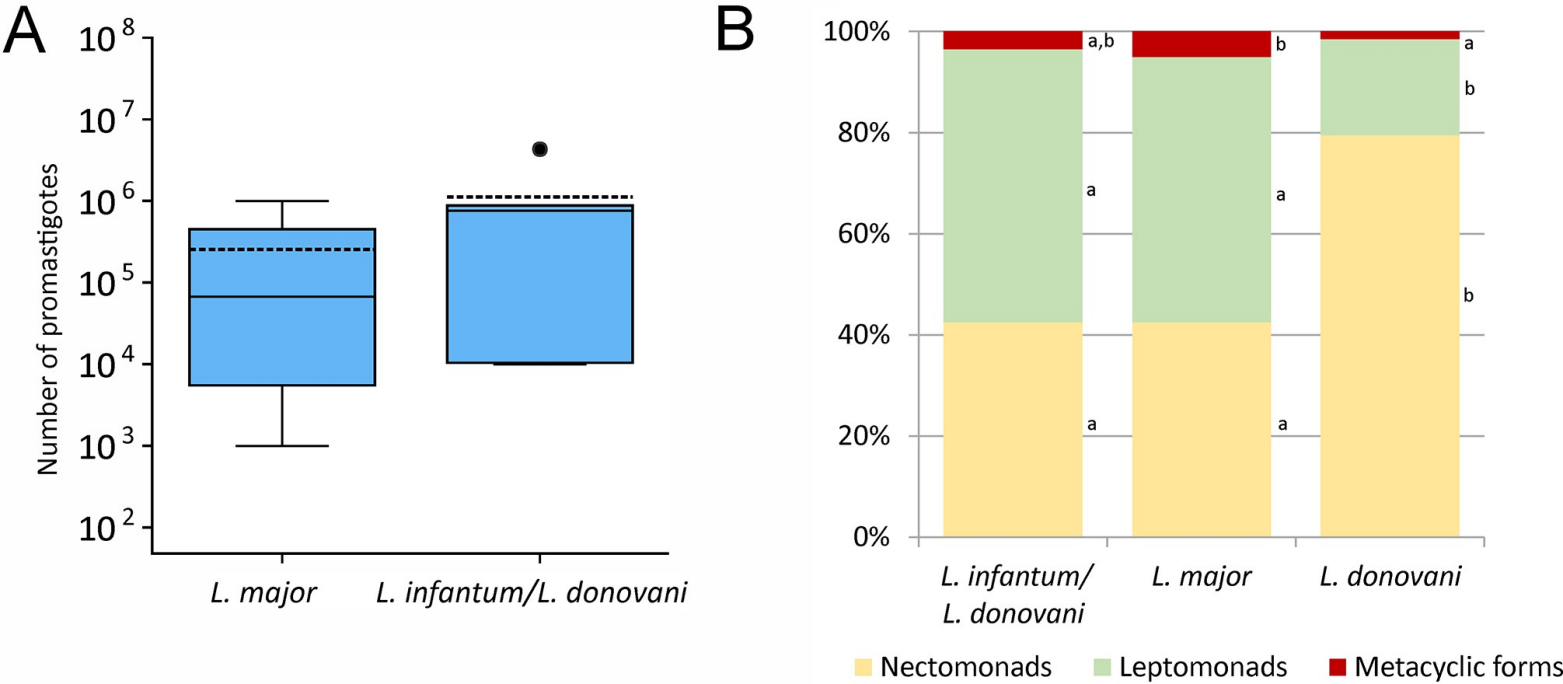
Numbers and morphology of *Leishmania* promastigotes in midguts of *P*. *tobbi* on day 10 PBM. A, Parasite load in sand flies infected with *L*. *major* (N = 14) and *L*. *infantum/donovani* hybrid CUK3 (N = 6); the original data available in S1 Dataset. In the boxplots, the box is bordered by upper and lower quartile (IQR, interquartile range), the horizontal full line denotes the median value, the horizontal dashed line shows the mean, whiskers denote 1.5 times IQR and circles denote outliers. B, Representation of morphological forms in 200 promastigotes of each species. Small letters to the right of the columns indicate the significance of differences in the representation of morphological forms: if the letters are the same, the representation of forms does not differ for these *Leishmania* species; the original data available in S2 Dataset.

Quantitative PCR showed that parasite loads on day 10 PBM were similar in *L*. *major* and control strain CUK3 (Fig 4A); the difference in median values and distribution was not significant (Nonparametric Median Test P = 0.628; Kruskal-Wallis Test P = 0.099). In *L*. *donovani*, the numbers of infected females were not sufficient to perform this analysis. Both *Leishmania major* and *L*. *donovani* formed metacyclic stages in *P*. *tobbi* females (Fig 4B). The difference in the representation of metacyclic forms in the two species was not significantly different from the control CUK3, but *L*. *major* formed significantly more metacyclics than *L*. *donovani* (P < 0.001, d.f. = 4, Χ^2^ = 74.431).

Both strains of *L*. *martiniquensis* developed in *P*. *tobbi* in a similar manner to that previously observed in *P*. *perniciosus*. On the second day PBM, promastigotes were enclosed within the peritrophic matrix and infection rates (84.4% of females infected with Cu2 strain and 76.7% of females infected with Aig strain) did not differ significantly between groups (P = 0.409, Χ^2^ = 1.767) (Fig 3C and 3D). However, differences in infection rates between *L*. *martiniquensis* and the control CUK3 strain later became highly significant (P < 0.001, Χ^2^ = 78.184 on day 4, P < 0.001, Χ^2^ = 69.263 on day 10). Control strain CUK3 produced heavy mature infections with colonization of the stomodeal valve, while Aig strain was not detected in any females and promastigotes of Cu2 strain were observed in 2 females on day 4 PBM and 1 female on day 10 PBM, but all these females had delayed defecation and low numbers of promastigotes survived in blood remnants in the abdominal midgut (Fig 3C and 3D). Thus, similarly to *P perniciosus*, *P*. *tobbi* cannot be considered a competent vector of *L*. *martiniquensis*.

### 3.3. Development of *L*. *donovani* in *P*. *argentipes* and *P*. *perniciosus*

**Fig 5:**
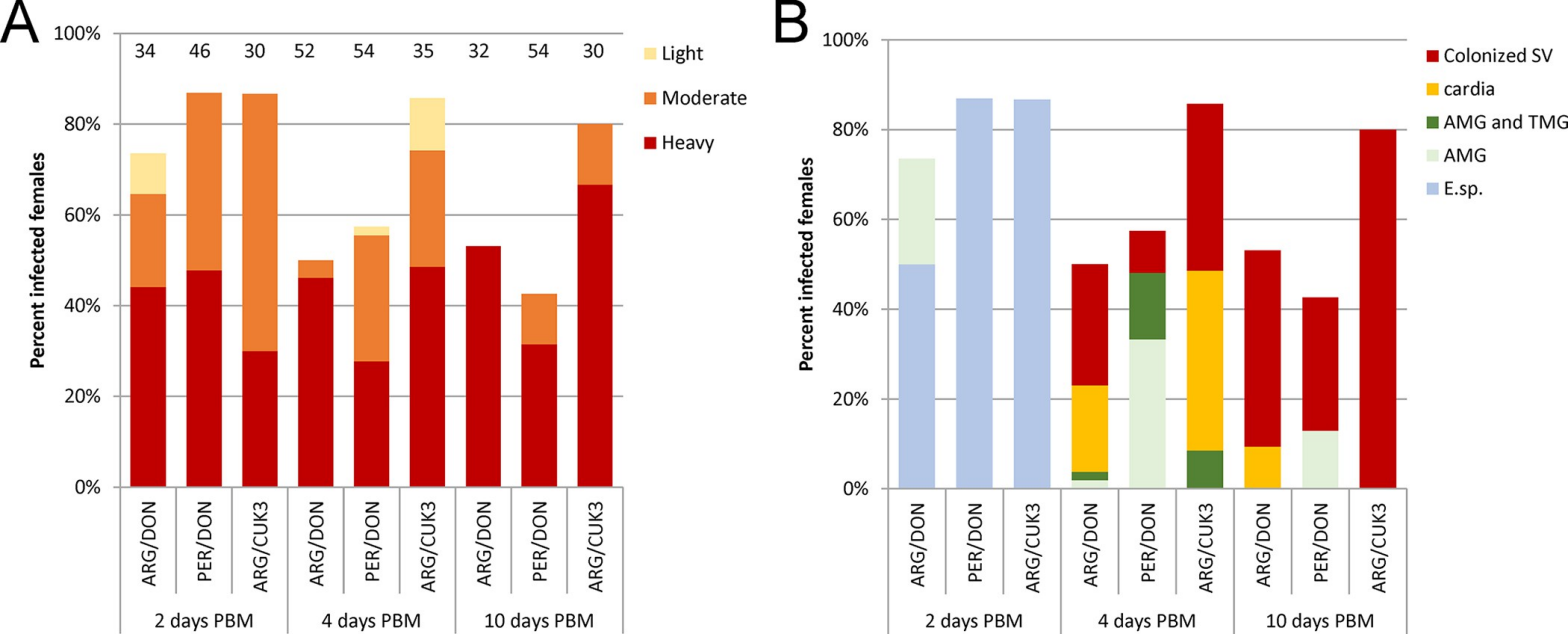
*Leishmania donovani* infections in *P*. *perniciosus* and *P*. *argentipes*. Summary of 2 experiments with *L*. *donovani* (DON) and *L*. *infantum/donovani* hybrid CUK3. A, intensities of infections; B, localisation of infections. The numbers of females examined are shown above the columns of infection intensities. SV, stomodeal valve; AMG, abdominal midgut; TMG, thoracic midgut; E. Sp., endoperitrophic space.

The low proportion of females infected with the Nepalese strain of *L*. *donovani* in both *P*. *perniciosus* and *P*. *tobbi* was surprising, so we conducted further experiments to compare the development of this strain with that in its natural Asian vector, *P*. *argentipes*. In addition, strain CUK3 (a hybrid of *L*. *infantum* and *L*. *donovani*), which was used as a control in *P*. *tobbi* (Section 3.2), was also included for comparison. On the second day PBM, infection rates were not significantly different (Fig 5A; P = 0.245, d.f. = 2, Χ^2^ = 2.812). In all groups, parasites colonized the stomodeal valve as early as day 4 PBM, and heavy mature infections with colonization of the stomodeal valve predominated on day 10 PBM (Fig 5B). However, from day 4 PBM, the difference between the Nepalese *L*. *donovani* and CUK3 strain in infection rates began to appear. The Nepalese strain produced low infection rates in both *P*. *argentipes* and *P*. *perniciosus*, significantly lower compared to the CUK3 strain in *P*. *argentipes* (P = 0.002, d.f. = 2, Χ^2^ = 12,541 on day 4 PBM, P = 0.004, d.f. = 2, Χ^2^ = 10.986 on day 10 PBM).

## 4. Discussion

Leishmaniases are traditionally regarded as diseases of tropical and subtropical regions. In southern Europe, only *L*. *infantum* is endemic, except for isolated cases of *L*. *tropica* in Greece [6–7]. However, given the emerging risk of new *Leishmania* species in Europe [4–5,37] it is important to investigate how successfully European sand fly species can support their transmission.

*Phlebotomus perniciosus* and *P*. *tobbi* are the main vectors of *L*. *infantum* in south-western and south-eastern Europe, respectively, and permissive vectors capable of supporting the development of *L*. *tropica* [28–29]. Our study showed that both *P*. *perniciosus* and *P*. *tobbi* support very well also the development of the Libyan strain of *L*. *major*. The percentages of infected females were fully comparable to the control group infected with *L*. *infantum* and in both species, strong mature infections with colonization of the stomodeal valve strongly prevailed and metacyclic forms were present on day 10 PBM. In contrast, previously studied Israeli *L*. *major* strain showed heavy infections in only about 20% of infected *P*. *tobbi* females and only 11% had the stomodeal valve colonized [30].

Our results also showed that the Asian strain of *L*. *donovani* from Nepal can produce heavy infections and colonize the stomodeal valve in *P*. *perniciosus* and *P*. *tobbi*. However, the infection rates in mature infections were low while Sadlova et al. (2011) [38] and Seblova et al. (2015) [30] reported previously high infection rates in *P*. *perniciosus* females infected with Ethiopian strains of *L*. *donovani*. We tested the hypothesis that the Nepalese strain is less compatible with the European sand fly species and would develop well in the natural Asian vector *P*. *argentipes*, but no significant difference was found between its infection rates in *P*. *perniciosus* and *P*. *argentipes*, whereas the Turkish hybrid *L*. *infantum/L*. *donovani* achieved significantly higher infection rate in *P*. *argentipes* in the same experiment. It cannot be excluded that the phenotype observed for the Nepalese strain in vectors is related to the history of this strain in the laboratories. Although it was isolated more recently than, for example, the Libyan strain of *L*. *major*, during *in-vitro* cultivation it may have undergone "bottle-neck" associated with impaired ability to interact with the vector. Therefore, these results suggest that testing at least two strains of parasites is optimal to assess vector competence.

The relatively low proportion of metacyclic promastigotes in the gut smears of most *Leishmania*–vector combinations, including controls, can be explained by the fact that the smears were taken from the whole gut and not only from the anterior (thoracic) part where metacyclic stages accumulate. The predominance of non-metacyclic forms in these samples is common even in natural vectors. For example, less than 5% of metacyclic promastigotes were detected in *P*. *argentipes* infected either with promastigotes or amastigotes of *L*. *donovani*, evaluated by the same method. Nevertheless, transmission efficiency was 42% and 56% in promastigote-infected and amastigote-infected groups, respectively [39]. Moreover, it has recently been shown that host infections can be initiated not exclusively by metacyclic stages, as long assumed, but also by haptomonads [40]. Haptomonads are stages with a very short flagellum with an enlarged flagellar tip attached to the cuticle of the stomodeal valve, important for blocking female feeding to facilitate the transmission of metacyclic stages to the host [41]. According to the most recent findings, haptomonads can detach [42] and directly contribute to the initiation of infection [40]. Thus, colonization of the stomodeal valve by haptomonads should be considered a key feature of vector competence.

The excellent development of the Libyan *L*. *major* in Spanish *P*. *perniciosus* and Turkish *P*. *tobbi* or the Turkish *L*. *infantum/L*. *donovani* hybrid in Indian *P*. *argentipes* suggest that the viability of *Leishmania* strains in vectors is not directly related to the geographical origin of the parasite. Another example of this phenomenon is the development of various strains of *L*. *major* in two proven vector species [43]–the strains differing in geographical origin and virulence showed very similar pattern of the development in both Turkish *P*. *papatasi* and Senegalese *P*. *duboscqi*. Similarly, two very different strains of *L*. *tropica*, one from a zoonotic focus in Israel and the second from an urban focus in Turkey, showed very similar development in Spanish *P*. *perniciosus* and Turkish *P*. *tobbi* [29]. However, the situation is probably different when considering *Leishmania* relationship with the vertebrate host. The Sub-Saharan *L*. *major* strain was maintained in three African rodent species for several months and these hosts were infectious to sand flies by xenodiagnosis, while the strain from the Middle East produced only weak infections [44]. In another study, different *L*. *major* strains developed well in their natural rodent hosts, but the African or Middle Eastern strains did not survive in Central Asian rodent *Rhombomys opimus* while no signs of disease were seen in African *P*. *obesus* infected with the Central Asian strain of the parasite [45]. Thus, virulence for a particular host seems to be related to the geographical origin of the parasite. For the spread of new *Leishmania* species to Europe, access to a suitable host may be more critical than access to a suitable vector.

The epidemiological context of *L*. *martiniquensis* in Europe is different from other *Leishmania* species. This member of the subgenus *Mundinia* has been recorded sporadically in a large geographical range from the Americas to Asia (reviewed by [46]). Interestingly, this *Leishmania* has also been reported in Central Europe: since 2009 it has been reported in cattle and horses in Germany [16], Switzerland [17], Austria, and the Czechia (recent Austrian and Czech findings in horses have not yet been published). The vectors of *L*. *martiniquensis* are not known, but at least in Central Europe they are not sand flies–these insects are rare in Germany, Switzerland and Austria and have not been recorded further north, as in the Czechia [37]. This is consistent with our results—neither *P*. *perniciosus* nor *P*. *tobbi* support the development of *L*. *martiniquensis*. Poor survival of *L*. *martiniquensis* in *P*. *argentipes* was described previously [47], only one strain out of four tested developed heavy mature infections in 7% of females. On the other hand, three *L*. *martiniquensis* strains showed excellent development in American biting midge *C*. *sonorensis* and two of them were transmitted by *C*. *sonorensis* bites to the mammalian host. Moreover, the other four tested species of *Mundinia* also developed significantly better in the biting midges than in sand flies [47]. Therefore, biting midges (Diptera: Ceratopogonidae) are suspected vectors of *L*. *martiniquensis* as well as other *Mundinia* species. This hypothesis is strongly supported by findings of *Mundinia*-infected biting midges in nature, in Thailand [48–50] and especially in Australia, where microscopic observations confirmed mature *L*. *macropodum* infections in midges of the genus *Forcipomyia* [51].

Thus, we can conclude that *P*. *perniciosus* and *P*. *tobbi* have all the prerequisites to serve as vectors of *L*. *major* and *L*. *donovani* in Europe, the definitive proof will be the demonstration of transmission of these parasites to the vertebrate host, which is our next objective. Further studies will of course be needed to identify European vectors of *L*. *martiniquensis* and to test the vector competence of other European sand fly species for *L*. *major*, *L*. *donovani*, *L*. *tropica* and *L*. *infantum*, whose expansion into other European countries can be expected. Last but not least, equally important is research on potential reservoir hosts for *L*. *martiniquensis*, as these are not known yet.

## Supporting information

S1 Dataset Promastigote numbers in sand fly guts. (XLSX)

S2 Dataset Measurements of *Leishmania* promastigotes from gut smears. (XLSX)
